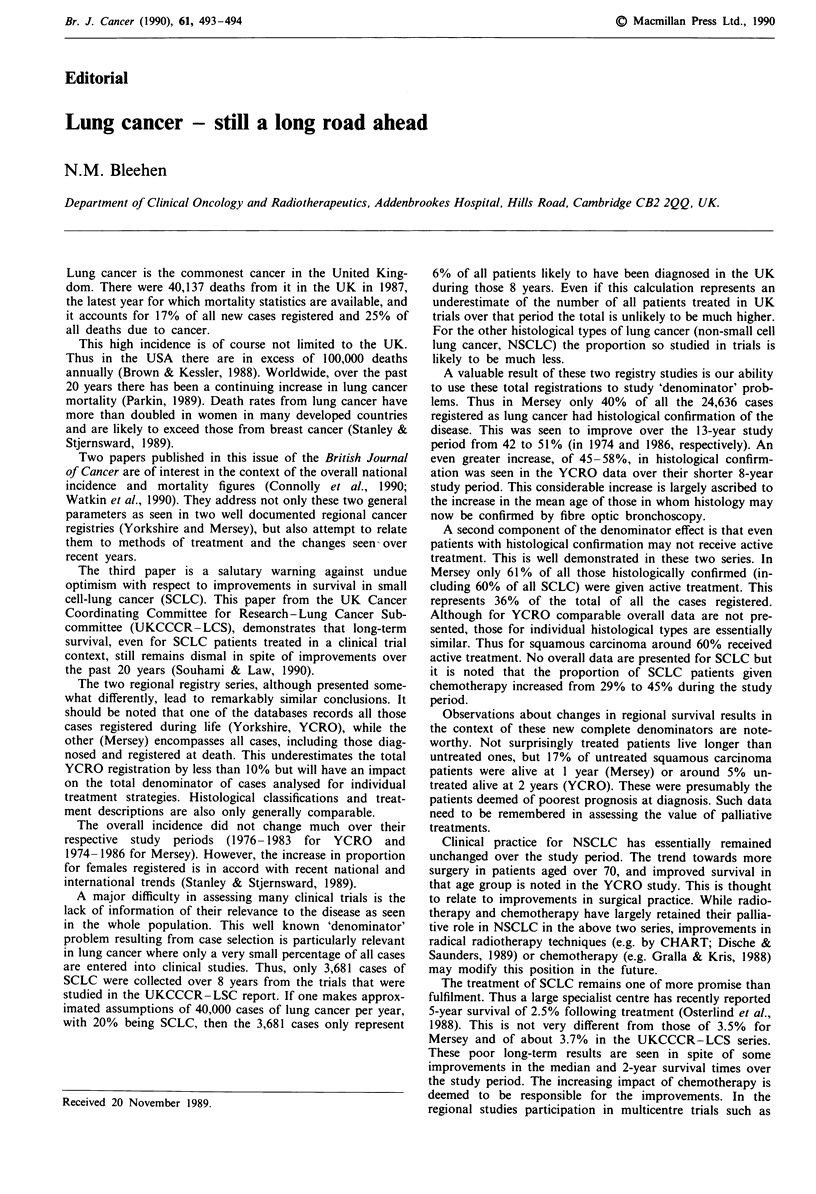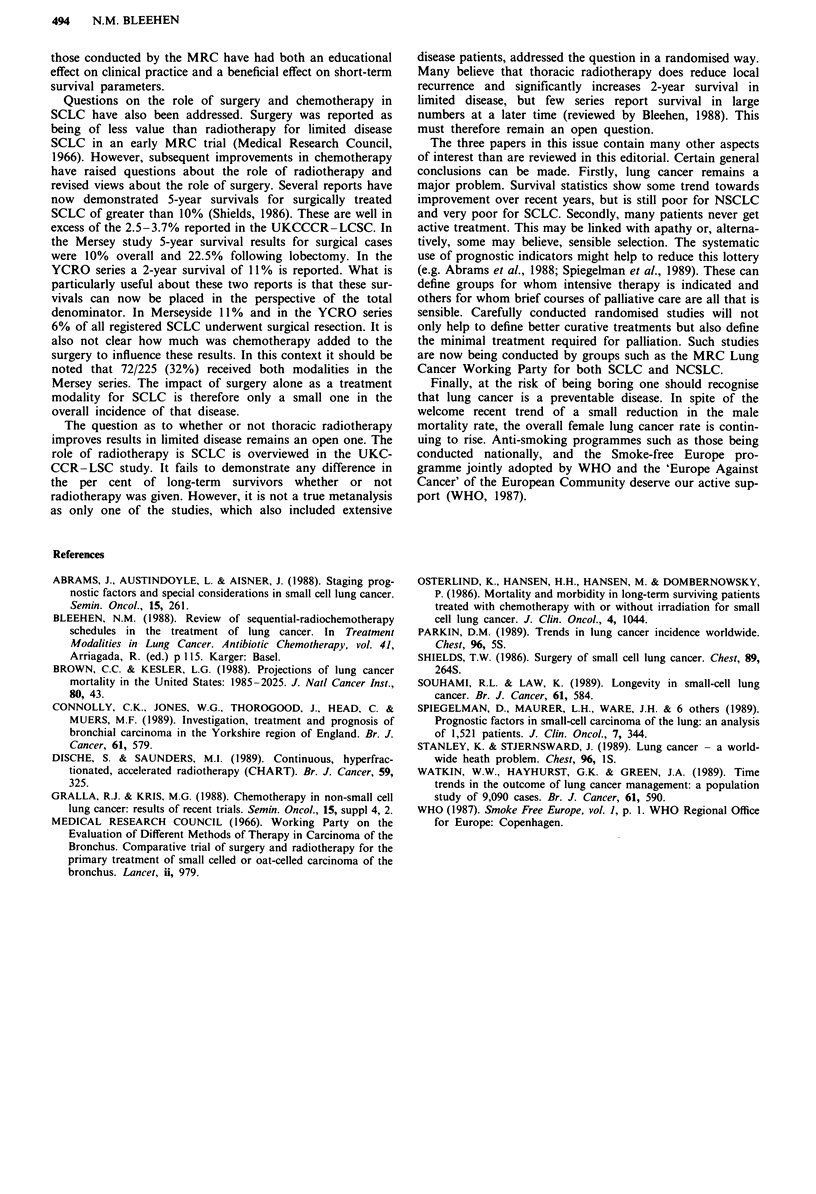# Lung cancer--still a long road ahead.

**DOI:** 10.1038/bjc.1990.110

**Published:** 1990-04

**Authors:** N. M. Bleehen

**Affiliations:** Department of Clinical Oncology and Radiotherapeutics, Addenbrookes Hospital, Cambridge, UK.


Br. J. Cancer (1990), 61, 493-494 © Macmillan Press Ltd., 1990

### Editorial

### Lung cancer – still a long road ahead

N.M. Bleehen

*Department of Clinical Oncology and Radiotherapeutics, Addenbrookes Hospital, Hills Road, Cambridge CB2 2QQ, UK.*

Lung cancer is the commonest cancer in the United Kingdom. There were 40,137 deaths from it in the UK in 1987, the latest year for which mortality statistics are available, and it accounts for 17% of all new cases registered and 25% of all deaths due to cancer.

This high incidence is of course not limited to the UK. Thus in the USA there are in excess of 100,000 deaths annually (Brown & Kessler, 1988). Worldwide, over the past 20 years there has been a continuing increase in lung cancer mortality (Parkin, 1989). Death rates from lung cancer have more than doubled in women in many developed countries and are likely to exceed those from breast cancer (Stanley & Stjernsward, 1989).

Two papers published in this issue of the *British Journal of Cancer* are of interest in the context of the overall national incidence and mortality figures (Connolly *et al.*, 1990; Watkin *et al.*, 1990). They address not only these two general parameters as seen in two well documented regional cancer registries (Yorkshire and Mersey), but also attempt to relate them to methods of treatment and the changes seen over recent years.

The third paper is a salutary warning against undue optimism with respect to improvements in survival in small cell-lung cancer (SCLC). This paper from the UK Cancer Coordinating Committee for Research-Lung Cancer Subcommittee (UKCCCR-LCS), demonstrates that long-term survival, even for SCLC patients treated in a clinical trial context, still remains dismal in spite of improvements over the past 20 years (Souhami & Law, 1990).

The two regional registry series, although presented somewhat differently, lead to remarkably similar conclusions. It should be noted that one of the databases records all those cases registered during life (Yorkshire, YCRO), while the other (Mersey) encompasses all cases, including those diagnosed and registered at death. This underestimates the total YCRO registration by less than 10% but will have an impact on the total denominator of cases analysed for individual treatment strategies. Histological classifications and treatment descriptions are also only generally comparable.

The overall incidence did not change much over their respective study periods (1976-1983 for YCRO and 1974-1986 for Mersey). However, the increase in proportion for females registered is in accord with recent national and international trends (Stanley & Stjernsward, 1989).

A major difficulty in assessing many clinical trials is the lack of information of their relevance to the disease as seen in the whole population. This well known 'denominator' problem resulting from case selection is particularly relevant in lung cancer where only a very small percentage of all cases are entered into clinical studies. Thus, only 3,681 cases of SCLC were collected over 8 years from the trials that were studied in the UKCCCR-LSC report. If one makes approximated assumptions of 40,000 cases of lung cancer per year, with 20% being SCLC, then the 3,681 cases only represent 6% of all patients likely to have been diagnosed in the UK during those 8 years. Even if this calculation represents an underestimate of the number of all patients treated in UK trials over that period the total is unlikely to be much higher. For the other histological types of lung cancer (non-small cell lung cancer, NSCLC) the proportion so studied in trials is likely to be much less.

A valuable result of these two registry studies is our ability to use these total registrations to study 'denominator' problems. Thus in Mersey only 40% of all the 24,636 cases registered as lung cancer had histological confirmation of the disease. This was seen to improve over the 13-year study period from 42 to 51% (in 1974 and 1986, respectively). An even greater increase, of 45-58%, in histological confirmation was seen in the YCRO data over their shorter 8-year study period. This considerable increase is largely ascribed to the increase in the mean age of those in whom histology may now be confirmed by fibre optic bronchoscopy.

A second component of the denominator effect is that even patients with histological confirmation may not receive active treatment. This is well demonstrated in these two series. In Mersey only 61% of all those histologically confirmed (including 60% of all SCLC) were given active treatment. This represents 36% of the total of all the cases registered. Although for YCRO comparable overall data are not presented, those for individual histological types are essentially similar. Thus for squamous carcinoma around 60% received active treatment. No overall data are presented for SCLC but it is noted that the proportion of SCLC patients given chemotherapy increased from 29% to 45% during the study period.

Observations about changes in regional survival results in the context of these new complete denominators are noteworthy. Not surprisingly treated patients live longer than untreated ones, but 17% of untreated squamous carcinoma patients were alive at 1 year (Mersey) or around 5% untreated alive at 2 years (YCRO). These were presumably the patients deemed of poorest prognosis at diagnosis. Such data need to be remembered in assessing the value of palliative treatments.

Clinical practice for NSCLC has essentially remained unchanged over the study period. The trend towards more surgery in patients aged over 70, and improved survival in that age group is noted in the YCRO study. This is thought to relate to improvements in surgical practice. While radiotherapy and chemotherapy have largely retained their palliative role in NSCLC in the above two series, improvements in radical radiotherapy techniques (e.g. by CHART; Dische & Saunders, 1989) or chemotherapy (e.g. Gralla & Kris, 1988) may modify this position in the future.

The treatment of SCLC remains one of more promise than fulfilment. Thus a large specialist centre has recently reported 5-year survival of 2.5% following treatment (Osterlind *et al.*, 1988). This is not very different from those of 3.5% for Mersey and of about 3.7% in the UKCCCR-LCS series. These poor long-term results are seen in spite of some improvements in the median and 2-year survival times over the study period. The increasing impact of chemotherapy is deemed to be responsible for the improvements. In the regional studies participation in multicentre trials such as

Received 20 November 1989.

those conducted by the MRC have had both an educational effect on clinical practice and a beneficial effect on short-term survival parameters.

Questions on the role of surgery and chemotherapy in SCLC have also been addressed. Surgery was reported as being of less value than radiotherapy for limited disease SCLC in an early MRC trial (Medical Research Council, 1966). However, subsequent improvements in chemotherapy have raised questions about the role of radiotherapy and revised views about the role of surgery. Several reports have now demonstrated 5-year survivals for surgically treated SCLC of greater than 10% (Shields, 1986). These are well in excess of the 2.5-3.7% reported in the UKCCCR-LCSC. In the Mersey study 5-year survival results for surgical cases were 10% overall and 22.5% following lobectomy. In the YCRO series a 2-year survival of 11% is reported. What is particularly useful about these two reports is that these survivals can now be placed in the perspective of the total denominator. In Merseyside 11% and in the YCRO series 6% of all registered SCLC underwent surgical resection. It is also not clear how much was chemotherapy added to the surgery to influence these results. In this context it should be noted that 72/225 (32%) received both modalities in the Mersey series. The impact of surgery alone as a treatment modality for SCLC is therefore only a small one in the overall incidence of that disease.

The question as to whether or not thoracic radiotherapy improves results in limited disease remains an open one. The role of radiotherapy is SCLC is overviewed in the UKCCCR-LSC study. It fails to demonstrate any difference in the per cent of long-term survivors whether or not radiotherapy was given. However, it is not a true metanalysis as only one of the studies, which also included extensive disease patients, addressed the question in a randomised way. Many believe that thoracic radiotherapy does reduce local recurrence and significantly increases 2-year survival in limited disease, but few series report survival in large numbers at a later time (reviewed by Bleehen, 1988). This must therefore remain an open question.

The three papers in this issue contain many other aspects of interest than are reviewed in this editorial. Certain general conclusions can be made. Firstly, lung cancer remains a major problem. Survival statistics show some trend towards improvement over recent years, but is still poor for NSCLC and very poor for SCLC. Secondly, many patients never get active treatment. This may be linked with apathy or, alternatively, some may believe, sensible selection. The systematic use of prognostic indicators might help to reduce this lottery (e.g. Abrams *et al.*, 1988; Spiegelman *et al.*, 1989). These can define groups for whom intensive therapy is indicated and others for whom brief courses of palliative care are all that is sensible. Carefully conducted randomised studies will not only help to define better curative treatments but also define the minimal treatment required for palliation. Such studies are now being conducted by groups such as the MRC Lung Cancer Working Party for both SCLC and NCSLC.

Finally, at the risk of being boring one should recognise that lung cancer is a preventable disease. In spite of the welcome recent trend of a small reduction in the male mortality rate, the overall female lung cancer rate is continuing to rise. Anti-smoking programmes such as those being conducted nationally, and the Smoke-free Europe programme jointly adopted by WHO and the 'Europe Against Cancer' of the European Community deserve our active support (WHO, 1987).

### References

ABRAMS, J., AUSTINDOYLE, L. & AISNER, J. (1988). Staging prognostic factors and special considerations in small cell lung cancer. *Semin. Oncol.*, **15**, 261.

BLEEHEN, N.M. (1988). Review of sequential-radiochemotherapy schedules in the treatment of lung cancer. In *Treatment Modalities in Lung Cancer. Antibiotic Chemotherapy, vol. 41*, Arriagada, R. (ed.) p 115. Karger: Basel.

BROWN, C.C. & KESLER, L.G. (1988). Projections of lung cancer mortality in the United States: 1985-2025. *J. Natl Cancer Inst.*, **80**, 43.

CONNOLLY, C.K., JONES, W.G., THOROGOOD, J., HEAD, C. & MUERS, M.F. (1989). Investigation, treatment and prognosis of bronchial carcinoma in the Yorkshire region of England. *Br. J. Cancer*, **61**, 579.

DISCHE, S. & SAUNDERS, M.I. (1989). Continuous, hyperfractionated, accelerated radiotherapy (CHART). *Br. J. Cancer*, **59**, 325.

GRALLA, R.J. & KRIS, M.G. (1988). Chemotherapy in non-small cell lung cancer: results of recent trials. *Semin. Oncol.*, **15**, suppl 4, 2.

MEDICAL RESEARCH COUNCIL (1966). Working Party on the Evaluation of Different Methods of Therapy in Carcinoma of the Bronchus. Comparative trial of surgery and radiotherapy for the primary treatment of small celled or oat-celled carcinoma of the bronchus. *Lancet*, **ii**, 979.

OSTERLIND, K., HANSEN, H.H., HANSEN, M. & DOMBERNOWSKY, P. (1986). Mortality and morbidity in long-term surviving patients treated with chemotherapy with or without irradiation for small cell lung cancer. *J. Clin. Oncol.*, **4**, 1044.

PARKIN, D.M. (1989). Trends in lung cancer incidence worldwide. *Chest*, **96**, 5S.

SHIELDS, T.W. (1986). Surgery of small cell lung cancer. *Chest*, **89**, 264S.

SOUHAMI, R.L. & LAW, K. (1989). Longevity in small-cell lung cancer. *Br. J. Cancer*, **61**, 584.

SPIEGELMAN, D., MAURER, L.H., WARE, J.H. & 6 others (1989). Prognostic factors in small-cell carcinoma of the lung: an analysis of 1,521 patients. *J. Clin. Oncol.*, **7**, 344.

STANLEY, K. & STJERNSWARD, J. (1989). Lung cancer – a worldwide heath problem. *Chest*, **96**, 1S.

WATKIN, W.W., HAYHURST, G.K. & GREEN, J.A. (1989). Time trends in the outcome of lung cancer management: a population study of 9,090 cases. *Br. J. Cancer*, **61**, 590.

WHO (1987). *Smoke Free Europe, vol. 1*, p. 1. WHO Regional Office for Europe: Copenhagen.